# Supplementary material for: Patterns of Intron Gain and Loss in Fungi
Source: PLoS Biol. 2004 Nov 30;2(12):e422. doi: 10.1371/journal.pbio.0020422 (PMC532390; doi:10.1371/journal.pbio.0020422)
Supplement: Table S1 — Also available at http://genes.mit.edu/NielsenEtAl/. (4.3 MB ZIP). [file pbio.0020422.st001.zip › NielsenEtAl/html/542.html]

AN7994.1.NCU01171.1.MG08646.1.FG06777.1


```
 CLUSTAL W (1.82) Multiple Sequence Alignments - Introns Inserted


Sequence 1: NCU01171.1	724 aa
Sequence 2: FG06777.1	721 aa
Sequence 3: MG08646.1	720 aa
Sequence 4: AN7994.1	724 aa
Alignment Length: 732 aa
Number Identitical Residues: 464 aa
Alignment Score (without introns) 21002


MG08646.1 	MDRQSAFSVHAFS---SNYEQGEDSNVQIQEQLLQFILDFRLENKFIYR2DQLRENALLG
NCU01171.1	MDYQSVFSARTYAGAASSYVPEEDSNQHIRAQLEAFILDFRLDNSFIYR2DQLRENALLK
FG06777.1 	MDRGSVYSAHVYE---PSFGENGDTRLQLQTQLETFILDFRLDNNFVYR2DQLRENALLK
AN7994.1  	MDRRTPYTLSVLA---PSTDGADESRTQIQSKLREFVLEFQLDNAFIYR2DQLRQNVLVK
          	**  : ::  .     ..     ::. ::: :*  *:*:*:*:* *:** ****:*.*: 

MG08646.1 	AYYCDVNIGDLIKFNEELAHRIVTEPTEIIPL0FEKALRKATQRIVYPHKQRIDLPEHQL
NCU01171.1	KYYCDVNIGDLIKFNEEIAHRLVTEPAEIIPL~FEAALKRCTHRIVFPNEPKIDLPDHQL
FG06777.1 	RYFCDVNINDLISFNEELAHRLASEPAEIIPL0FENALKKCTHRIVFPHEPKIEIPEHQL
AN7994.1  	HYYCDIDIAHLISYNEELAHKLTTEPADIIPL0FEAALQQCTQRIVYPSQRDIVLPTHQL
          	 *:**::* .**.:***:**::.:**::**** ** **::.*:***:* :  * :* ***

MG08646.1 	LLHSNEEDVSIRKLDSMTISRLVRVPGIVIGASVMSSKANSLTIQCRNCGHTTDIPVSGG
NCU01171.1	LLHSNAEDVSIRNLDSLTISRLVRVPGIVIGASVMSSKATELHVQCRNCGHVQHVHVSGG
FG06777.1 	LLHSNADDVSIRHLDSETISRLVRVPGIVIGASVMSSKATELHIQCRNCGHTQNIPVLGG
AN7994.1  	LLHSSATHISIRDLNATNISHLVRIPGIVIGASTISSKATTVHIRCKSCDHAENIRVEGG
          	****.  .:***.*:: .**:***:********.:****. : ::*:.*.*. .: * **

MG08646.1 	FTGVTLPRRCERNKGPVD-SSDKCPLDPYFVQHEKSQFVDQQVIKLQEAPDDVPVGELPR
NCU01171.1	FSGATLPRTCGRIRAPGD-PGEKCPMDPYFVQHEKSRFVDQQIIKLQEAPDQVPVGELPR
FG06777.1 	FTGVTLPRQCSRSRVPND-PTPKCPMDPYFVAHEKSRFVDQQIIKLQEAPDQVPVGELPR
AN7994.1  	FSGLTLPRRCGRERQPGEEPDTQCPLDPYVVAHEKSQFVDQQVLKLQEAPDQVPVGELPR
          	*:* **** * * : * :..  :**:***.* ****:*****::*******:********

MG08646.1 	HVLISADRYLTNRVVPGSRCTIMGIFSIYQNKGSKSSTSG-AVAIRTPYLRAVGIQTDID
NCU01171.1	HVLISADRYLTNRVVPGSRCTVMGIFSIYQSGGNKKSTSG-AVAIRTPYLRAVGIQTDID
FG06777.1 	HVLISADRYLTNRVVPGSRCTVMGIFSIYQNKASKNSSTGGAVAIRTPYLRAVGIQSDID
AN7994.1  	HVLISADRYLANRVVPGSRCTVMGIFSIYQKGGKKD---G-AVAIRNPYLRAVGITTDLD
          	**********:**********:********. ..*.   * *****.******** :*:*

MG08646.1 	TASRGNATFSPEEEQEFLEMSRREDIYKVLAGCIAPSIYGNADIKKAILCLLFGGSKKIL
NCU01171.1	QTAKGLAVFSEEEEQEFLELSRRPDLYNVMAECIAPSIYGNKDIKKAILCLLMGGSKKIL
FG06777.1 	QAAKGNATFSEEEEQEFLEMSRRPDIYDVMTDCIAPSIYGNRDIKRSILCLLLGGSKKIL
AN7994.1  	HTAKGSYIFSEEEEQEFLELSRRPDLYEALARSIAPSIYGNLDIKKAIVCLLMGGSKKIL
          	 :::*   ** ********:*** *:*..:: .******** ***::*:***:*******

MG08646.1 	PDGMKLRGDINVLLLGDPGTAKSQLLKFVEKVAPIAIYTSGKGSSAAGLTASVQRDQSTK
NCU01171.1	PDGMRLRGDINVLMLGDPGTAKSQLLKFVEKVAPIAIYTSGKGSSAAGLTASVQRDASTR
FG06777.1 	PDGMRLRGDINVLLLGDPGTAKSQLLKFVEKAAPISIYTSGKGSSAAGLTASVQRDQSTR
AN7994.1  	PDGMKLRGDINVLLLGDPGTAKSQLLKFTEKVSPIAIYTSGKGSSAAGLTASVQRDPATR
          	****:********:**************.**.:**:******************** :*:

MG08646.1 	EFYLEGGAMVLADNGVVCIDEFDKMRDEDRVAIHEAMEQQTISIAKAGITTILNARTSVL
NCU01171.1	EFYLEGGAMVLADGGVVCIDEFDKMRDEDRVAIHEAMEQQTISIAKAGITTILNARTSVL
FG06777.1 	EFYLEGGAMVLADGGVVCIDEFDKMRDEDRVAIHEAMEQQTISIAKAGITTILNARTSVL
AN7994.1  	EFYLEGGAMVLADGGVVCIDEFDKMRDEDRVAIHEAMEQQTISIAKAGITTILNSRTSVL
          	*************.****************************************:*****

MG08646.1 	AAANPIFGRYDELKSPGENIDFQTTILSRFDMIFIVRDEHEPGKDQRIAKHVMSLHQGRT
NCU01171.1	AAANPIFGRYDDMKTPGENIDFQTTILSRFDMIFIVKDEHERGRDERIAKHVMGIHMGGR
FG06777.1 	AAANPIFGRYDDMKTPGENIDFQTTILSRFDMIYIVKDEHSREKDETMAKHVLGIQMNGR
AN7994.1  	AAANPIFGRYDDLKTPGENIDFQTTILSRFDMIFVVRDDHERSRDENIARHVMGVHMGGR
          	***********::*:******************::*:*:*.  :*: :*:**:.:: .  

MG08646.1 	NVQEQVESEIPFDKLRRYISYCKS2RCAPRLSAEAAERLSSHFVTIRRQVHAAEMEANTR
NCU01171.1	GVEERIEAEIPVEKMRRYISYCRS2RCAPRLSPEAAEKLSSHFVAIRKQVHASELEANAR
FG06777.1 	GTEDMTESEIPIDKMRRYITYCKT2RCAPRLSPEAAEKLSSHFVSIRRQVHAAEIEANSR
AN7994.1  	GMEEQVEAEIPLEKMKRYISYCRS~RCAPRLSPEAAEKLSSHFVSIRKQVHRAELDANAR
          	. ::  *:***.:*::***:**:: *******.****:******:**:*** :*::**:*

MG08646.1 	SSIPITVRQLEAIVRITESLAKMQLAPIATEDHVKEAIRLFLASTLDAVNQGADQGS-RE
NCU01171.1	SSIPITVRQLEAIVRITESLAKLTLSPVATEKHVDEAIRLFLCSTMDAVNQGGSTGSNRE
FG06777.1 	SSIPITVRQLEAIVRITESLAKLTLSPIATEVHVDEAIRLFLCSTMDAVNQGSNQGS-RE
AN7994.1  	SSIPITVRQLEAIVRITESLAKLSLQPIATEAHVDEAIRLFLASTMDAITQGEGQGS-RE
          	**********************: * *:*** **.*******.**:**:.** . ** **

MG08646.1 	INDEASKVEAELRRRLPIGWSTSFATLRREMVEGKGFSEMALNRALKILQRRETIMFRNQ
NCU01171.1	LNEETSRVEAELKRRLPIGWSTSLATLRREMVEGKGFSEVALNRALMMLQRRDTIMFRNQ
FG06777.1 	LNDEVNRLEAELKRRLPIGWSTSLSTLKKEMVEGKGYSEQALNRALMVLQRRDTIMFRNQ
AN7994.1  	MMEEVSKIEDELKRRLPIGWSTSLATLRREFVDGRGYTEQALNRAVIVLQRRGTIQIRSG
          	: :*..::* **:**********::**::*:*:*:*::* *****: :**** ** :*. 

MG08646.1 	GAQ~IYRNGA------
NCU01171.1	GAQ~VYRNGA------
FG06777.1 	GAQ~VYRNGA------
AN7994.1  	GSQ0HRNDTLVYAQRG
          	*:*   .:    :. .
```
